# Semipersistently Transmitted, Phloem Limited Plant Viruses Are Inoculated during the First Subphase of Intracellular Stylet Penetrations in Phloem Cells

**DOI:** 10.3390/v13010137

**Published:** 2021-01-19

**Authors:** Jaime Jiménez, Aránzazu Moreno, Alberto Fereres

**Affiliations:** Instituto de Ciencias Agrarias, Consejo Superior de Investigaciones Científicas (ICA-CSIC), C/Serrano 115 dpdo, 28006 Madrid, Spain; jaime.jimenez@ufl.edu (J.J.); amoreno@ica.csic.es (A.M.)

**Keywords:** *Beet yellows virus*, *Myzus persicae*, *Closterovirus*, phloem-pd, electrical penetration graphs

## Abstract

The green peach aphid *Myzus persicae* Sulzer is the main vector of the semipersistently transmitted and phloem-limited *Beet yellows virus* (BYV, *Closterovirus*). Studies monitoring the *M. persicae* probing behavior by using the Electrical penetration graphs (EPG) technique revealed that inoculation of BYV occurs during unique brief intracellular punctures (phloem-pds) produced in companion and/or sieve element cells. Intracellular stylet punctures (or pds) are subdivided in three subphases (II-1, II-2 and II-3), which have been related to the delivery or uptake of non-phloem limited viruses transmitted in a non-persistent or semipersistent manner. As opposed to non-phloem limited viruses, the specific pd subphase(s) involved in the successful delivery of phloem limited viruses by aphids remain unknown. Therefore, we monitored the feeding process of BYV-carrying *M. persicae* individuals in sugar beet plants by the EPG technique and the feeding process was artificially terminated at each phloem-pd subphase. Results revealed that aphids that only performed the subphase II-1 of the phloem-pd transmitted BYV at similar efficiency than those allowed to perform subphase II-2 or the complete phloem-pd. This result suggests that BYV inoculation occurs during the first subphase of the phloem-pd. The specific transmission mechanisms involved in BYV delivery in phloem cells are discussed.

## 1. Introduction

Aphids are the major vectors of noncirculatively (NC) transmitted viruses, a virus category characterized by no latent period and loss after molting [1]. Within NC viruses, two groups of viruses have been commonly established: the nonpersistently (NP) and semipersistently (SP) transmitted [2]. Since the pioneer study conducted by [3], numerous studies have focused on the feeding behavior activities of sap-sucking insects associated with virus transmission. The electrical penetration graph (EPG) technique [4] has been key in the study of different aphid stylet activities involved in the transmission of plant viruses [5,6]. The different aphid stylet activities in plants have been correlated with several characteristic EPG patterns. The dominant EPG waveform during pathway phase is waveform C, which is correlated with the intercellular stylet pathway periodically interrupted by brief intracellular punctures that are identified in EPG recordings as potential drops (‘pds’) [7].

The inoculation of stylet-borne NC viruses (non-persistently and semipersistently transmitted) occurs during ‘pds’ produced by the aphid primarily in epidermal or mesophyll cells [6,8]. These intracellular punctures are composed of three different phases (I, II and III). Within phase II, three subphases are distinguished. Whereas subphase II-1 is associated with injection of watery saliva into the cell and inoculation of non-persistently-transmitted virus particles [6,9], subphase II-3 is associated with uptake of cell cytoplasm and therefore acquisition of non-persistent viruses [6,10]. Subphase II-2 but not subphase II-1 was associated with the inoculation of the semipersistently transmitted *Cauliflower mosaic virus* (CaMV, *Caulimovirus*) by *Brevycorine brassicae* [8]. However, the specific activity linked to the transmission of CaMV occurring along this subphase II-2 (salivation and/or egestion) remains unknown.

Within the semipersistently transmitted virus group, certain viruses such as *Beet yellows virus* (BYV, *Closterovirus*) are restricted to the phloem tissues of the host plant. BYV virus particles are composed of a flexuous filamentous single-stranded RNA of approximately 1300 nm in length and 12 nm in diameter [11,12]. Failed attempts to acquire purified BYV virions by aphids from solution have led to suggestions that BYV need the presence of a helper protein to bind to the aphid cuticle [13,14], however this has not been proofed [15]. In fact, other viruses within family *Closteroviridae,* bind to the vector by simple interaction of the minor coat protein [16,17]. The transmission of the whitefly-transmitted *Lettuce infectious yellows* virus (LIYV, *Closterovirus*) is determined by a minor coat protein (CPm) retention mechanism in the anterior foregut of its vector [16]. Also, *Citrus tristeza virus* (CTV, *Closterovirus*) has been suggested to bind to the *N*-acetylglucosamine (NAG) moieties of the cuticular surface of its vector *Toxoptera citricida* via the CPm, in addition to p61 and p65 heat shock proteins [17]. In case of BYV, efficient assembly by CPm requires homologous Hsp70h, p64 and p20 proteins, with these proteins also likely playing a role in virus transmission in addition to the CPm [18].

There is considerable information about BYV transmission process by its main aphid vector *Myzus persicae* is available. Whereas BYV acquisition is optimized after long times of sap phloem ingestion, BYV infection occurs very efficiently after unique intracellular punctures (phloem-pds) produced by *M. persicae* in phloem cells (sieve elements and companion cells) [19,20]. These particular potential drops in phloem cells are distinct from the standard-pds produced in non-vascular tissues and are always preceding the phloem sieve element salivation phase (E1 waveform). Also, these brief intracellular punctures in the phloem have been associated with the inoculation of persistently transmitted, phloem limited Luteoviruses [21]. Nevertheless, the specific subphase(s) of the phloem-pd involved in virus inoculation as well as the specific stylet activities associated to the delivery of virions from the aphid cuticle to the plant cell remain unknown.

Here, we studied the behavioral aspects linked to the transmission of semipersistently transmitted, phloem limited viruses during the three distinct phloem-pd subphases by conducting real-time artificially ended EPG recordings using viruliferous *M. persicae* carrying BYV on sugar beet test plants.

## 2. Materials and Methods

### 2.1. Plants, Aphid and Virus Maintenance

A colony of *M. persicae* Sulzer (Mp89 clone) was used in the experiments. The colony was started from a single virginiparous female collected from a pepper plant at El Encín (Madrid, Spain) in 1989 and later maintained on *Capsicum annuum* cv. ‘Luesia’. Later, the same *M. persicae* colony was maintained in *Beta vulgaris* cv. ‘Julietta’ since 2015. Aphid colonies were maintained in a growth chamber at temperatures of 22:18 °C (light/dark) using a 16:8 h (light/dark) photoperiod.

A BYV isolate PV-0981 (Leibniz-Institut, Plant Virus Collection, Braunschweig, Germany) was maintained in *B. vulgaris* cv. ‘Julietta’ by sequential passages using *M. persicae* as vector. Source plants for transmission tests were used 4–6 weeks after BYV infection after checking their infection status by DAS-ELISA test [22]. New BYV-infected plants were generated by placing groups of 10 *M. persicae* adults for an acquisition access period (IAP) of 24 h on an BYV-infected plants and later transferred to a 10–12 day-old sugar beet for an inoculation access period of 24 h. Source plants were maintained in a growth chamber at temperatures of 24/20 °C (light/dark) using photoperiods of 16/8 h (light/dark).

Test plants used for EPG experiments were also *B. vulgaris* (cv. ‘Julietta’) at two emerging true leaf stage. Before experiments, test plants were maintained in a virus-free growth chamber at temperatures of 24/20 °C (light/dark) using photoperiods of 16/8 h (light/dark). After EPGs, test plants were sprayed with Confidor 20 LS (Bayer CropScience, Leverkusen, Germany (100 ppm active ingredient [ai]) after aphid exposure and kept in a greenhouse under natural light conditions and at 23 ± 4 °C for 4 weeks. Virus infection in test plants was doubly checked by first visualization of symptoms and later verification by serological detection by DAS-ELISA test [22].

### 2.2. EPG Setup and Transmission Tests

**Figure 1:**
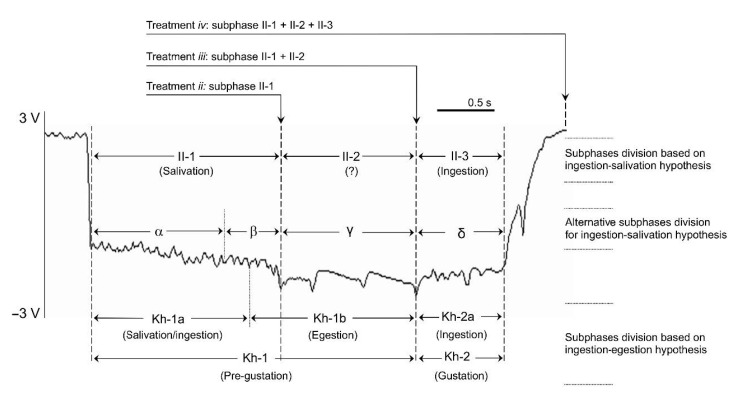
Example of an EPG signal recorded during the stylet intracellular puncture in the sieve elements or companion cells (phloem-pd) produced by *Myzus persicae* on sugar beet. The different treatments applied for the study of BYV inoculation and the pd subphase division according to ingestion-salivation hypothesis (I-SH) and ingestion-egestion hypothesis (I-EH) are indicated. Proponents of the I-SH divide the pd into subphases II-l, II-2, and II-3 [6]. We based our current study on that commonly accepted hypothesis, interrupting the pd during the II-1 of the phloem-pd (treatment *ii*), during the II-2 of the phloem-pd (treatment *iii*) and a just after a single complete phloem-pd was produced by the aphid (treatment *iv*). An alternative subphase division has been proposed in agreement with the I-SH, subdividing the pd into additional subphases based on signal features [23]. Subphase II-1 is subdivided into α and β, with subphase II-2 named as γ and II-3 subdivided into δ and ε (ε is not indicated here as phloem-pd does not include this particular subphase). In the I-EH [24,25], the pd is divided into pregustation (Kh-1) and gustation (Kh-2) phases. Similar intraphase comparisons of waveforms, subdivide phases Kh-1 and Kh-2 into subphases or waveform types: Kh-la and Kh-lb, and Kh-2a and Kh-2b, respectively (Kh-2b is not indicated here as phloem-pd does not include this particular subphase). *Y*-axis: EPG output voltage expressed in volts (V); *X*-axis: EPG recording time expressed in seconds (s).

To determine the particular pd subphase associated with BYV delivery, aphid stylets penetrations were monitored and recorded by using a DC-EPG (GIGA-8; EPG Systems) device [4], connected to a USB AD card (DI-710; DATAQ Instruments) and a PC laptop. Signals were acquired and analyzed using Stylet+ software for Windows (EPG Systems). Newly emerged viruliferous *M. persicae* apterous adults (2–3 day-old) were used for EPG experiments. Aphids were collected from the virus-free colony and allowed to acquire the virus for an AAP of 24 h on a 4–6 week-old BYV-infected sugar beet. After the AAP, viruliferous aphids were collected and wired to a gold wire connected to a copper electrode. EPG experiments were conducted on a two-emerging true leaf stage (10–12-day-old) sugar beets. Immediately, aphids were connected to the EPG device to monitor the IAP on the healthy sugar beet test plants (Plant A). The feeding process was artificially terminated by carefully lifting the gold wire during/after real time observation of the following patterns (Figure 1): (*i*) after a complete standard-pd, (*ii*) during subphase II-1 of a phloem-pd, (*iii*) during subphase II-2 of a phloem-pd, (*iv*) after a complete phloem-pd (subphases II-1, II-2 and II-3). Real-time identification of a phloem-pd and its specific subphases was made according to the criteria explained below.

For EPG recordings manually ended during the subphase II-2 (treatment *iii*) and just after the complete phloem-pd (treatment *iv*), the phloem-pd was distinguished due to the unique subphase II-2 observed for this type of pd, significantly lower in number and frequency of intervals from the standard-pd [19,20]. However, for EPG recordings interrupted during the subphase II-1 of the phloem-pd (treatment *ii*), the only strategy to identify the phloem-pd in vivo was through the potential drop magnitude, another distinguishable feature observed in the phloem-pd, also significantly lower than the potential drop magnitude measured for the standard-pd [19,20]. Therefore, to interrupt in vivo the subphase II-1 of the phloem-pd, the following method was followed. EPG waveforms produced by aphids were observed and, after a series of consecutive standard-pd and irregular C waveform, *M. persicae* produce a steady waveform C (usually voltage differences of no more than ~2 volts between the highest and lowest peak observed along this waveform). The time needed to observe that kind of steady waveform C is quite variable, occurring sometimes after 5 standard pds, but others after more than 30 standard-pds. During that steady waveform C, the potential drop magnitude of the standard-pds produced by the aphids was marked by placing a scotch tape on the monitor screen just below the potential level observed within a standard-pd. Then, to identify a phloem-pd the aphid was carefully and quickly lifted up and the probe was interrupted once a drop in voltage of less magnitude well above the marked voltage level was observed.

After an EPG recording was successfully terminated, the aphid was allowed to feed for an IAP of 24 h on a second clean receptor plant (Plant B), without monitoring the behavior, in order to check its ability to acquire and transmit the virus under optimal conditions (a single aphid was used per each test plant). Aphids that were unable to infect either A or B test plants were discarded from the analysis.

### 2.3. Determining Phloem-pd Occurrence

After conducting the EPGs coupled to the virus transmission tests, each recording was later analyzed with Stylet + a software in order to confirm the criteria initially stablished for each treatment. The main patterns for the identification of a phloem-pd are the unique EPG pattern performed by the aphid along subphase II-2 together with the comparison of the potential drop magnitude with previous standard-pd produced by the aphid [19,20]. Therefore, for distinguishing the experimental treatments concerning the phloem-pd (*ii, iii, and iv*) indicated above, these two criteria were applied for an accurate phloem-pd identification. However, for treatment *ii*, phloem-pd occurrence could be only determined on the basis of the potential drop magnitude comparison. Previous studies studying the role of the phloem-pd in the transmission of BYV revealed an average potential drop magnitude for the phloem-pd of 84.3% in comparison with the previous standard-pd [19]. Therefore, all recordings were thoroughly analyzed and potential drop of the phloem-pd was measured and compared with the potential drop magnitude of the previous standard-pd produced by the aphid. For treatment *ii*, aphids were considered to produce a phloem-pd when the voltage drop magnitude of the interrupted subphase II-1 was lower than 84.3% of the total potential drop magnitude measured in the previous standard pd.

### 2.4. Duration of Aphid Stylet Phloem Intrusion

Phloem activities produced by the aphids resulting in successful BYV inoculation were thoroughly inspected in order to investigate the duration of aphid stylet intrusion in comparison with BYV transmission. Therefore, duration of each phloem-pd subphase was measured within the three treatments involving phloem activities (*ii*, *iii* and *iv*). In order to study an accurate comparison between subphase duration leading to BYV infection, only recordings of aphids resulting in infection in plant A (or both test plants A + B) were analyzed. Having measured all times of phloem intrusion, a minimum time for inoculation of closteroviruses could be determined on the basis of BYV transmission results derived from EPG experiments for each particular treatment. Therefore, the total duration of the subphase II-1 (treatment *ii*), subphases II-1 and II-2 (treatment *iii*) and subphases II-1, II-2 and II-3 (treatment *iv*) performed by the aphids was measured for each recording.

Once the key subphase of the phloem-pd involved in BYV transmission were determined in EPG our studies, further studies were performed in order to investigate BYV transmission during this particular subphase. Therefore, duration of subphase II-1 of the EPG recordings from aphids leading to BYV infection in either A or A + B test plants were studied and compared to those viruliferous aphids that did not infect test plant A but infected plant B. Also, to investigate correlations between the duration of stylet phloem intrusion and the success of BYV inoculation, similar time intervals (3 groups including 0.3 s each) were stablished in subphase II-1. BYV transmission efficiencies occurring at each of the time intervals were compared.

### 2.5. Statistical Analysis

Transmission rates of BYV obtained for each specific EPG treatment and at different intervals of time within subphase II-1 were compared by a Monte Carlo χ^2^ Pearson test, with Bonferroni correction. Comparisons between phloem-pd subphases duration and duration of the subphase II-1 of the phloem-pd between aphids leading to infection in plant A and those that did not, were compared by performing a Student’s *t* test. Potential drop magnitude (∆V) and the frequency of intervals in subphase II-2 (Ints/s) were compared between standard and phloem-pds using a Student’s paired *t* test or a Wilcoxon test depending on the frequency distribution of the data. If the raw data were normal, then Student’s paired *t* test was use; if neither the raw data nor any of the transformations were normal, then the non-parametric Wilcoxon test was used. Non-gaussian variables were transformed applying ‘ln (x + 1)’ or ‘sqrt (x + 1)’ transformations. All analyses were run using the SPSS 25.0.0.1 package [26].

## 3. Results

### 3.1. Aphid Stylet Activities and BYV Transmission

EPG-assisted transmission experiments showed that BYV transmission occurred from the onset of the phloem-pd produced by *M. persicae*. Aphids transmitted BYV at an efficiency of 56% (9/16) on recordings interrupted during the subphase II-1 of the phloem pd, with no significant differences with BYV transmission efficiency obtained after the interruption of the phloem-pd during the subphase II-2 (71%; 10/14) or after a complete phloem-pd (69%; 11/16) (Table 1). No BYV transmission was obtained when aphids were allowed to produce a single standard-pd (0/18) before any phloem-pd was produced (Table 1).

### 3.2. Duration of Phloem-pd Subphases

**Figure 2:**
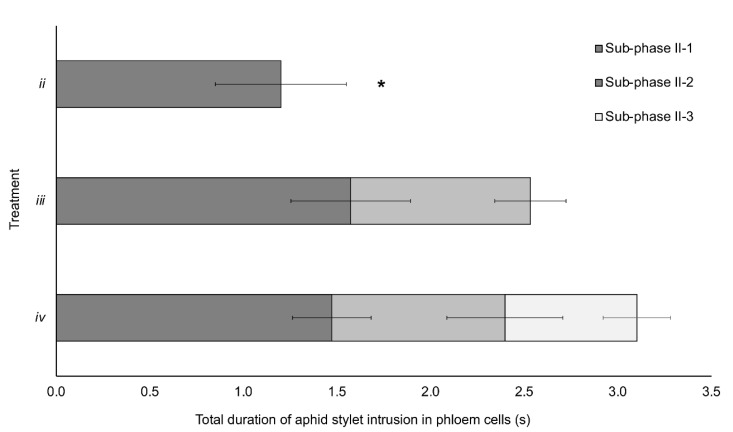
Duration of the different phloem-pd subphases in the three treatments from EPG recordings of aphids producing BYV infection during recorded acquisition access period by EPG (either plant A or both A and B plants). For treatment *ii* (*n* = 9), only subphase II-1 was recorded; for treatment *iii* (*n* = 10), both subphases II-1 and II-2 were recorded, and the three subphases (II-1, II-2 and II-3) were recorded in treatment *iv* (*n* = 11) (complete phloem-pd). Asterisk stands for significant differences according to a Student’s *t* test (*p* < 0.05).

Mean duration of the different subphases and the accumulated time of stylet intrusion in phloem cells along the three treatments is represented in Figure 2. The mean duration of subphase II-1 was significantly shorter in treatment ii (1.2 s) in comparison with subphase II-1 in treatment iii (1.57 s; *p* = 0.019) and treatment iv (1.47 s; *p* = 0.024). No significant differences were observed when the comparison of duration of subphase II-1 between treatments iii and iv was conducted (*p* = 0.471). Duration of subphase II-2 was similar between treatments iii and iv (*p* = 0.769).

### 3.3. BYV Transmission in Association with the Duration of Subphase II-1

**Figure 3:**
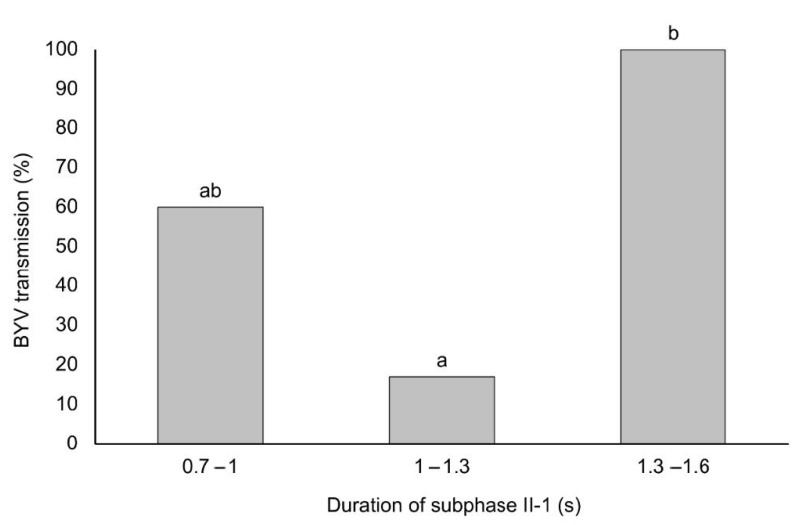
Transmission efficiency of BYV at different durations of subphase II-1 of the phloem-pd. EPG recording interrupted during the subphase II-1 were split and grouped according to intervals of 0.3 s: 0.7 to 1; 1 to 1.3; 1.3 to 1.6 s (*X*-axis). BYV transmission efficiency at each specific duration of subphase II-1 is also represented (*Y*-axis). Different letters show significant differences according to a Student’s *t* test (*p* < 0.05).

Once subphase II-1 was found to be the responsible for the delivery of BYV particles into the phloem cells, we studied the EPG recordings interrupted during the subphase II-1 (treatment *ii*) and measured and compared the duration of the subphase II-1 between EPG recordings from viruliferous aphids that did lead to BYV infection in either plant A or A + B and those that did not (only plant B was infected). Aphids that produced infection produced a longer subphase II-1 (1.20 ± 0.27 s) in phloem cells than aphids that did not produce infection (1.10 ± 0.20 s), but the differences were not statistically significant (*p* = 0.245) (Figure 3).

Also, the duration of II-1 subphase was divided in 3 classes (0.7–1 s; 1–1.3 s and 1.3–1.6 s) and BYV transmission efficiency was determined for each of the classes. Aphids resulted in significantly higher BYV transmission efficiency when the subphase II-1 lasted between 1.3–1.6 s (100%; 5/5), showing significant differences with those that produced a subphase II-1 between 1–1.3 s (17%; 1/6). Aphids interrupted at early stages of the subphase II-1 (0.7–1 s) transmitted BYV at an intermediate rate of 60% (3/5), with no significant differences with those aphids producing longer subphase II-1 (Figure 3).

### 3.4. Identification of the Phloem-pd

**Figure 4:**
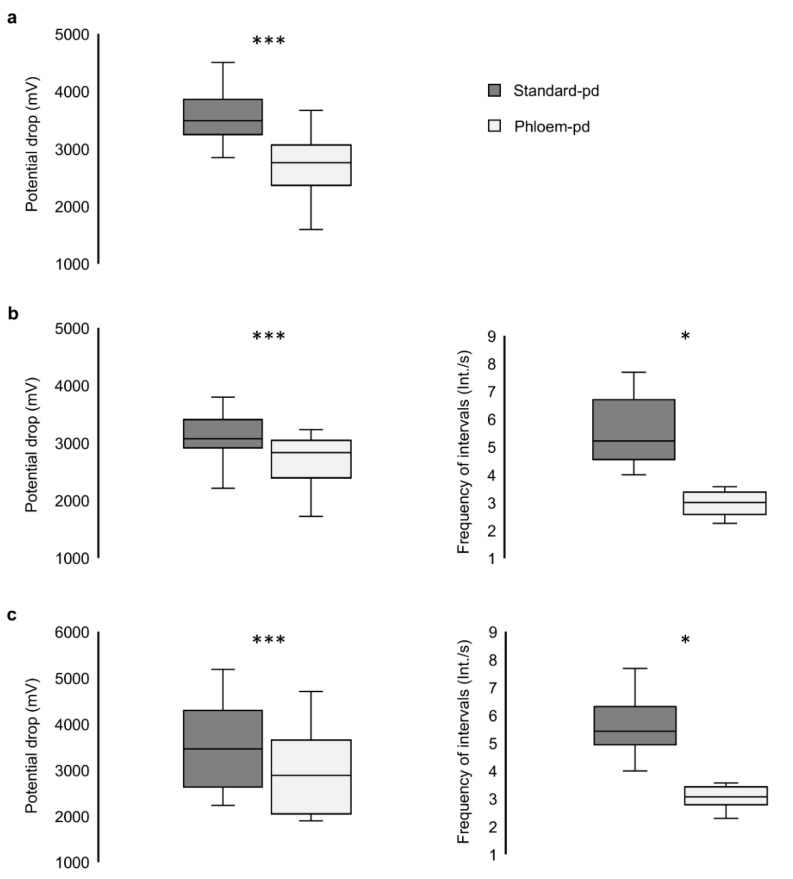
Comparison of the potential drop magnitude and frequency of intervals in subphase II-2 between standard and phloem-pd. The phloem-pd characteristics were compared with previous standard-pd along the recording of the three treatments including the phloem pd: (**a**) treatment *ii* (*n* = 16), (**b**) treatment *iii* (*n* = 14), and (**c**) treatment *iv* (*n* = 16). Asterisks stand for significant differences between variables according to a Student’s paired *t* test or a Wilcoxon test (***, *p* < 0.001; *, *p* < 0.05).

Analysis of the EPG patterns of the phloem-pd in our recordings fit the standards already described for this type of potential drop [19]. The comparison between the voltage drop magnitude of the phloem-pd with the previous standard-pd produced by the aphid revealed clear differences in the three treatments involving phloem contact (*p* < 0.001) (Figure 4). Also, the frequency of intervals was lower in the phloem pd in comparison with the standard-pd in the two treatments including this particular pd subphase (*p* = 0.035 for treatment *iii*; *p* = 0.015 for treatment *iv*) (Figure 4).

Complete information of recordings interrupted during subphase II-1 (treatment *ii*) is provided in Table 2. The study of all the pds produced by the aphids along the recording revealed a minimum of 4.0 int/s in the 16 recordings meeting the criteria previously established for a phloem-pd. The potential drop magnitude of the phloem-pd represented a mean value of 77.93% in comparison with the total potential drop magnitude measured in the previous standard-pd produced by the aphid. A minimum of 0.76 s of stylet tips intrusion into phloem cells was long enough to successfully inoculate BYV.

## 4. Discussion

Our results show unequivocal evidence that BYV particles are delivered by its vector *M. persicae* into the phloem cells of the host plant from the beginning of aphid stylet intrusion into phloem cells (either companion or sieve element cells). Results from our EPG experiments indicated that subphase II-1 of the phloem-pd is the one involved in the inoculation of BYV, a semipersistently transmitted, phloem limited virus. Brief intrusion (0.76–1.57 s) into phloem cells was enough to inoculate BYV at a maximum efficiency of transmission. Increased BYV transmission efficiencies obtained for treatments *iii* and *iv*–though no significant differences– may be explained by the fact that treatment *ii* often included an incomplete subphase II-1 since we manually disturbed the feeding during this subphase to avoid the transition to subphase II-2. On the contrary, for treatments *iii* and *iv*, aphids were allowed to produce a complete subphase II-1, explaining why it lasted significantly longer than in treatment *ii* (Figure 2).

These results we obtained for a semipersistently transmitted, phloem limited virus (BYV) in association to the three subphases of the potential drop produced by aphids differed from those already described for non-phloem limited semipersistent viruses (e.g., CaMV). In the case of CaMV, virus delivery/inoculation occurs exclusively within subphase II-2 of standard potential drops (standard-pds) produced by the aphid *Brevicoryne brassicae* in non-vascular tissues [8]. Moreover, CaMV was detected in a specific part of the common duct of its aphid vector, concretely in the ‘acrostyle’ [27]. Therefore, authors proposed two hypotheses to explain the delivery of CaMV particles into the plant during subphase II-2. The first hypothesis postulated the old ingestion–egestion hypothesis (I-EH) proposed by Harris [24] for viruses transmitted in a non-persistent manner; the second hypothesis dealing with an additional aphid salivation phase occurring during subphase II-2.

It is well known that salivation occurs during subphase II-1 [9] but other activities such as egestion could concomitantly occur at some point that would explain why BYV can be dislodged before the occurrence of subphase II-2 of a phloem-pd. Regurgitation or egestion of cytoplasm contents previously ingested by the aphid could accumulate in the food canal. At some point during subphase II-1 of the phloem-pd, aphids could dislodge BYV particles from the foregut or food canal and deliver them to the plant. The fact that most of EPG recordings that were terminated during the last past of subphase II-1 became infected (Figure 3) supports that egestion may start at the end of subphase II-1 and continue during subphase II-2. This would explain why there was an increase, although not statistically significant, in the transmission rate when aphid probes were interrupted during subphase II-2.

The I-EH hypothesis proposed by Harris and Harris [25], suggests that egestion would occur at the end of the subphase II-1 (referred as Kh-1b subphase; Figure 1). In fact, the Kh-1b subphase includes the complete subphase II-2 but also the very end of subphase II-1 (as defined by [28]). Moreover, the end of the subphase II-1 was further described by Tjallingii as β [23], due to its distinct pattern in comparison to the first part of subphase II-1 (so-called α) (Figure 1). Therefore, in the EPG recordings interrupted in late stages of subphase II-1, aphids would begin likely to produce subphase Kh-1b (or β pattern). Our results show that aphids that were interrupted at the late stage of subphase II-1 transmitted BYV particles at a higher rate than those interrupted at early or intermediate stages of the subphase II-1 of the phloem-pd. In fact, within the 16 recordings of viruliferous aphids interrupted during the subphase II-1, all of the 5 aphids that were interrupted after a long subphase II-1 (1.3–1.6 s; Figure 3) were able to transmit the virus (test plant A was positive). Thus, our results suggest that the subphase Kh-1b (egestion) is in fact involved in the transmission of BYV. We observed that BYV transmission efficiency increased in late stages of the subphase II-1 and also there was an increase in 15% of BYV transmission efficiency (although not significantly different) in EPG recordings interrupted during the subphase II-2 of the phloem-pd (Figure 1) due to in that treatment the whole Kh-1b subphase would be produced by the aphids. The fact that aphids still transmitted BYV at an efficiency of 60% (3/5) in the early stages of the subphase II-1 phloem-pd (0.7–1 s) could reflect inoculation by watery salivation in a similar manner as proposed in the salivation-ingestion hypothesis [6]. In fact, egestion during standard pds could dislodge some BYV particles retained in the foregut or food canal that would reach the common duct until a phloem pd is produced. Then, the egested particles could be inoculated by salivation during the beginning of subphase II-1 (α) of the phloem-pd. Thus, our results suggest that egestion together with salivation could be involved in the process of inoculation of BYV.

Finding out the specific retention sites of BYV within the aphid mouthparts would be key in order to confirm the precise mechanism(s) (either egestion, salivation or both) involved in BYV particle dislodging and further delivery into the phloem cells. Previous treatments with formalin and UV radiation in the vector suggested that this virus is not retained in the distal portion of the aphid stylets but at some point, at the anterior alimentary track [29]. More recently, the semipersistently transmitted and phloem-limited *Citrus tristeza virus* (CTV, *Closterovirus*) was detected in the foregut of *Toxoptera citricida* [17]. There is evidence that the anterior foregut or cibarium of the whitefly vector is the retention site of criniviruses [16]. Other semipersistently aphid-transmitted viruses such as *Anthriscus yellows virus* (AYV) and *Parsnip yellow fleck virus* (PYFV) were also found in the foregut of its aphid vector *Cavariella aegopodii* [30]. Thus, closteroviruses and the related criniviruses seem to be retained in the foregut of their insect vectors.

If BYV is retained in the foregut or food canal of *M. persicae*, then the old I-EH would explain the process of virus inoculation into plant cells [24]. On the contrary, if BYV is retained in the common duct of *M. persicae*, two hypotheses can be proposed for the subphase II-1 of the pd as responsible of BYV delivery. First, the salivation-ingestion hypothesis: BYV particles are flushed from the common duct during intracellular secretion of watery saliva, similar to the mechanism described for NP viruses [6,9]. However, for BYV, only the penetration of companion/sieve element cells by viruliferous aphids leads to systemic infection by the phloem-limited BYV. Secondly, the egestion hypothesis [24,25] could not be ruled out as responsible of virus delivery during the last part of subphase II-1. If this assumption is true, salivation at the beginning followed by egestion of previously ingested contents would occur during subphase II-1 and would dislodge the virus particles from their retention sites.

There is currently no experimental evidence that aphids egest during penetrations of superficial or phloem cells [31]. If egestion occurs in both subphases II-1 and II-2, or only in one of them is something that needs to be demonstrated. In terms of EPG signal, the main distinguishable pattern of the phloem-pd in comparison with the standard-pds is the different shape of subphase II-2. However, as observed in our experiments, the occurrence in subphase II-2 of the phloem-pd does not have any significantly influence in BYV inoculation efficiency and its different and unique shape in this subphase possibly is influenced by the distinct type of cell punctured (CC/SE complex for the phloem-pd). Whereas the II-2 is associated with salivation or egestion into the cell in the standard-pds (CaMV inoculation takes place during the subphase II-2), the subphase II-2 of the phloem-pd may actually represent a brief ingestion from phloem cells. In fact, subphase II-2 of the phloem pd resembles a very short E2 waveform in which the aphid could acquire content from phloem cells (in addition to the subphase II-3, already described as cytoplasm uptake activity [6]). In spite that aphid saliva is thought to prevent occlusion in compatible aphid-plant combinations [32], the phloem-pd likely represents a mode of very short phloem sampling in which any phloem plant defense mechanism could be triggered.

In summary, aphid stylet activities involved during subphase II-2 of the phloem-pd still remains unknown. However, according to our results, subphase II-1 of the phloem pd likely represents a combination of watery salivation followed by egestion of previously ingested sap that results in the inoculation of BYV. Further studies of the retention sites of BYV are needed for a better understanding of the aphid activity involved during subphase II-1 of the phloem-pd and confirm our findings. That would greatly contribute to the knowledge of plant virus transmission by aphids.

## Figures and Tables

**Table 1:** Relationship between *Myzus persicae* stylet activities in sugar beet and inoculation efficiency of *Beet yellows virus*.

Aphid Stylet Activities (EPG Waveforms Observed) ^a^	Total EPG Recordings	Discarded Data ^b^	Infected Plants		Total	% BYV Transmission Efficiency ^d^
			Plant A^+^ (A^+^ B^+^ or A^+^ B^−^) ^c^	Only Plant B^+^		
(i) A single complete standard-pd	47	29	0	18	0/18	0% a
(ii) Subphase II-1 of the phloem-pd	78	62	9	7	9/16	56% b
(iii) Subphase II-2 of the phloem-pd (subphases II-1 + II-2)	48	34	10	4	10/14	71% b
(iv) A single complete phloem-pd (subphases II-1 + II-2 + II-3)	50	34	11	5	11/16	69% b

**^a^** Viruliferous aphids were removed from test plants (1 aphid/test plant) after specific EPG waveform patterns were observed. **^b^** Discarded data include total number of EPG recordings ruled out due the presence of either a previous phloem-pd (treatment *ii*, *iii* and *iv*), a potential drop of the phloem-pd out of criterion (>84.3% potential drop magnitude of the previous standard-pd for treatment ii) or absence of infection in any of the A or B plants tested in the four treatments (non-viruliferous aphids). **^c^** Plant A was the plant where the aphid feeding was monitored until the production of the four waveform patterns was detected. Plant B was the plant to which aphids were transferred from plant A in order to assess the initial virus acquisition by the aphid. **^d^** Virus transmission efficiency was calculated by dividing the number of recordings where plant A became infected by the total number of recordings where either plant A or plant B or both test plants became infected (raw division represented on the left column ‘Total’). Different letters show significant differences according to a Monte Carlo χ^2^ test (Bonferroni correction) (*p* < 0.05).

**Table 2:** EPG recordings interrupted during the subphase II-1 of the phloem-pd. EPG recording were thoroughly inspected in order to discard the occurrence of a previous phloem-pd. Plants infected during EPG recording for each EPG recording are expressed. The total number of potential drops produced by the aphid along the recording as well as minimum frequency of intervals observed in a subphase II-2 are indicated. Moreover, percentage of potential drop magnitude of the phloem-pd in comparison with the previous standard-pd was measured in order to determine the production of the phloem-pd. Duration of each interrupted subphase II-1 was measured.

EPG Recording	Infected Plants	No. of Complete Standard Potential Drops	Lower Subphase II-2 Frequency (Int./s)	% ΔV Phloem-pd (mV)	Duration Subphase II-1 of the Phloem-pd (s)
1	Only A	37	4.17	69.24	1.34
2	Only A	47	5.0	84.19	0.98
3	A + B	29	5.15	67.82	1.12
4	A + B	34	4.34	73.93	1.36
5	A + B	26	5.26	67.74	1.45
6	A + B	46	4.0	72.33	1.31
7	A + B	28	4.29	82.17	0.76
8	A + B	44	4.35	81.98	0.93
9	A + B	58	4.55	81.47	1.57
10	Only B	73	4.17	79.33	0.78
11	Only B	42	4.35	83.53	1.27
12	Only B	29	5.88	74.86	1.19
13	Only B	42	4.55	77.54	0.87
14	Only B	26	4.55	83.43	1.2
15	Only B	51	4.42	84.25	1.09
16	Only B	24	5.41	83.04	1.28
Mean	-	39.75	4.65	77.93	1.16

## Data Availability

Data is contained within the article.
